# Immune Evasion Strategies of Pre-Erythrocytic Malaria Parasites

**DOI:** 10.1155/2014/362605

**Published:** 2014-05-07

**Authors:** Hong Zheng, Zhangping Tan, Wenyue Xu

**Affiliations:** Department of Pathogenic Biology, Third Military Medical University, Chongqing 400038, China

## Abstract

Malaria is a mosquito-borne infectious disease of humans. It begins with a bite from an infected female *Anopheles* mosquito and leads to the development of the pre-erythrocytic and blood stages. Blood-stage infection is the exclusive cause of clinical symptoms of malaria. In contrast, the pre-erythrocytic stage is clinically asymptomatic and could be an excellent target for preventive therapies. Although the robust host immune responses limit the development of the liver stage, malaria parasites have also evolved strategies to suppress host defenses at the pre-erythrocytic stage. This paper reviews the immune evasion strategies of malaria parasites at the pre-erythrocytic stage, which could provide us with potential targets to design prophylactic strategies against malaria.

## 1. Developmental Bottlenecks at the Pre-Erythrocytic Stage

After the infected female* Anopheles* mosquitoes bite and inject sporozoites into the host skin, the deposited sporozoites spread from the injection site within several hours [1]. Although approximately 20% of sporozoites are drained into the lymphatic system, most sporozoites enter the blood circulation [1]. To invade hepatocytes, sporozoites in the blood must safely cross the Kupffer cells (KCs), which are interspersed throughout the sinusoidal lining [2]. After passing through the sinusoidal cell layer, sporozoites traverse several hepatocytes until they ultimately settle in the final one. Inside the final hepatocyte, sporozoites are enclosed by a parasitophorous vacuole and develop into schizonts [3]. Finally, merozoites exit the hepatocyte in the form of merosomes [4].

During this process, sporozoites encounter host robust innate immune responses. Up to 20–30% of sporozoites entering the lymphatic system are impeded at the proximal lymph node, and most parasites are degraded within DCs [5]. It is still unknown about the sensor involved in this process, but our recent data suggested that sporozoite might be recognized by Toll-like receptor 2 (TLR2) on the innate immune cells, as sporozoite lysate could activate TLR2 and knockout of TLR2 significantly promoted the development of the exoerythrocyte form (unpublished data). Even inside the liver, hepatocyte damage during sporozoite transmigration releases DAMPs (damage-associated molecular pattern molecules) and triggers innate immune responses that suppress the pre-erythrocytic stage [6]. Very recently, exoerythrocyte form (EEF) RNA was reported to be recognized by Mda5 in hepatocyte, leading to the production of IFN-*α*/*β*, which triggered a type I IFN response in the innate immune cells to limit the development of liver-stage [7]. Consistently, innate immune cells, such as NK, *γδ*T, and CD4^−^ CD8^−^ NK1.1+ TCR*αβ* ^int^ cells, have been known to be activated and inhibit the development of intrahepatic parasites during primary infection [8–10]. By an unknown mechanism, the ongoing blood-stage infection induces the expression of the host iron regulatory hormone hepcidin, which impairs the growth of subsequently inoculated sporozoites [11]. These results indicated that innate immune responses create several bottlenecks that inhibit the development of sporozoites into EEF. Although an average of only 123 sporozoites is injected by the bite of a single infected mosquito, a successful infection can be established [12], suggesting that sporozoites overcome these bottlenecks. Growing evidence shows that sporozoites have developed several strategies to escape host defenses during the development of the pre-erythrocytic stage, and we will discuss this issue in the following text.

## 2. Concealed Sporozoites Resist Phagocytes and Develop in the Skin

The skin membrane barrier is one of the most important parts of innate immunity, acting as the first line of defense against invading organisms. However, biting mosquitoes liberate many different soluble components, such as antihistamines, vasodilators, anticoagulants, platelet aggregation inhibitors, and immunomodulators, from their salivary glands. All of these components assist in sporozoite survival and facilitate their inoculation.

After inoculation, sporozoites stay in the skin for several hours [1, 13] and are activated into a state of readiness for the hepatic stages after they shift from the mosquito to the mammalian host [14]. However, their capacity for migration allows sporozoites to avoid destruction by phagocytes and growth arrest by nonphagocytic cells in the host dermis. Some sporozoites that are deficient in cell migration, such as spect (sporozoite microneme protein essential for cell traversal)−/− or spect2−/− sporozoites, are immobilized in the dermis, associated with CD11b+ cells, and destroyed by phagocytes [15]. Interestingly, ~10% of sporozoites transform into EEFs within the epidermis or the dermis, especially in the immune-privileged hair follicles [16]. However, subsequent overwhelming data showed that EEFs developing in the skin may not lead to a blood-stage infection [17], and their development in the skin remained to be confirmed in human malaria infection. Most sporozoites leaving the injection site invade the dermal blood circulation and travel to the liver; some entering the lymphatic system are degraded inside the DCs after a short differentiation period [5]. However, it is unknown whether sporozoites are phagocytized by DCs or actively invade DCs; it is also unknown whether it is beneficial for the host to elicit immune responses to clear the parasites or for malaria parasites to suppress the host immune responses.

## 3. Suppression of the Function of Kupffer Cells by Sporozoites

Once inside the circulatory system, sporozoites rapidly reach the liver. Sporozoites, however, are initially arrested in the sinusoid by specific binding of the stellate cell-derived ECM (extracellular matrix) proteoglycans, which extend from the Disse space through EC (endothelial cell) fenestrations [18, 19]. To invade hepatocytes, sporozoites must cross the continuous cell layer lining the sinusoids. The arrested sporozoites then glide freely for several minutes along the sinusoidal endothelium until meeting a KC, the resident macrophage of the liver. Previous intravital microscopy and electron microscopy supported that sporozoites actively pass through KCs but not ECs, and the interaction of CSP (circum-sporozoite protein) with chondroitin and heparan sulfate proteoglycans on the surface of KCs allows entry to the liver parenchyma [2, 20]. However, multiplicity of sporozoite crossing mechanisms was revealed recently by using spinning-disk confocal imaging. It was found that most sporozoites penetrate the sinusoidal barrier through ECs (53%), and some specifically cross KCs (~24%). Some sporozoites can cross the gaps between ECs or between an EC and a KC, independent of their cell-transversal capacity. Thus, gap crossing may be observed for the cell crossing-deficient sporozoite mutants SPECT, SPECT2, and CelTOS (cell-traversal protein for ookinetes and sporozoites), which all induce a blood infection, though with reduced efficiency [21–24].

**Figure 1:**
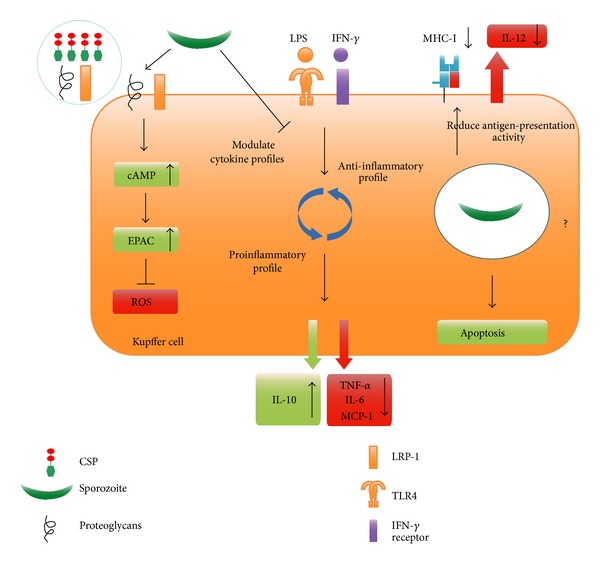
Sporozoites suppress the function of KCs. The binding of sporozoite CSP to proteoglycans and LRP-1 upregulates cAMP/EPAC and prevents the formation of ROS (left). Exposure to sporozoites downregulates the inflammatory cytokines TNF-*α*, IL-6, and MCP-1 and upregulates the anti-inflammatory cytokine IL-10 after stimulation with IFN-*γ* or LPS (middle). Sporozoite infection also downregulates MHC I and IL-12p40 and induces apoptosis in KCs (right).

It is puzzling that sporozoites safely traverse KCs, which provide innate immunity against microorganisms invading hepatocytes. The mechanisms responsible for this migration are becoming clearer. The binding of sporozoite CSP to the LRP-1 (low-density lipoprotein receptor-related protein) and proteoglycans on the KC surface increases the levels of intracellular cAMP/EPAC and prevents the formation of ROS (reactive oxygen species) [25]. Sporozoite contacting with KC also downregulates the inflammatory cytokines TNF-*α*, IL-6, and MCP-1 and upregulates the anti-inflammatory cytokine IL-10 after stimulation with IFN-*γ* or LPS [26]. In addition, the binding of sporozoites also induced KC apoptosis [26]. Further study found that the ability to migrate across cells is not only required for the malaria parasite to reach the liver [15], but also for its resistance to clearance by KCs, as sporozoites with high cell-crossing capacities kill KCs during this process [24]. In addition, the antigen-presentation activity of KCs, including the expression of MHC-I and IL-12, is severely reduced in mice challenged with sporozoites compared with those immunized with irradiation-attenuated sporozoites [27]. We previously showed that pretreatment with TLR agonists, especially CpG, significantly inhibits sporozoite development into EEF, potentially by enhancing the phagocytic capacity of KCs [28]; this result also suggested that sporozoites suppress KC function, and they could actively penetrate KCs if the phagocytic function of KCs is suppressed by sporozoites (Figure 1).

It is assumed that sporozoites traverse KCs without forming parasitophorous vacuoles [24]. However, previous study showed that sporozoites in KCs are isolated in parasitophorous vacuoles, which are formed to avoid lysosomal degradation [29]. CSP in parasitophorous vacuoles is released into the cytoplasm of host hepatocytes via its PEXEL domain [30] and inhibits host cell protein synthesis [31]. Therefore, it is interesting to investigate whether CSP could also suppress the function of KCs through inhibiting protein synthesis.

## 4. The Manipulation of Hepatocytes

After penetrating the sinusoidal cell layer, sporozoites invade hepatocytes and develop into EEFs. Unlike many other microbial organisms that utilize the phagocytic properties of their host cells for invasion, sporozoites actively invade hepatocytes. Sporozoites possibly use the cholesterol uptake pathway to invade hepatocytes. In addition to tetraspanin CD81 [32] and CD9 [33], the successful invasion of hepatocytes by sporozoites requires the host hepatocyte SR-BI (scavenger receptor BI) [34], which mediates the selective uptake of cholesteryl esters from both high- and low-density lipoprotein. However, sporozoites always pass through several hepatocytes prior to the final hepatocyte in which they develop [3]. Although the reason for this process is not well defined, it is likely that sporozoites choose the best environment for their differentiation into merozoites. The migration through hepatocytes increases sporozoite competency for differentiation by inducing the exocytosis of sporozoite apical organelles that are involved in the formation of an intracellular vacuole for infection [35]. The exocytosis of apical organelles during sporozoite migration is mediated by the malaria parasite adenylyl cyclase *α* and cAMP signaling [36]. Hepatocyte damage caused by transmigration is essential for making neighboring hepatocytes more susceptible to early parasite development; this process occurs by the activation of a HGF (hepatocyte growth factor)/cMET-dependent pathway and reorganization of host cell actin cytoskeleton [37]. However, SPECT1- [23] or SPECT2-defective [22] sporozoites, which cannot cross cells, infect hepatocytes* in vitro*, suggesting that transmigration may not be indispensable for the development of sporozoites in hepatocytes. Migration and invasion are two different sporozoite phenotypes that are regulated by the interaction of the sporozoite main surface protein CSP [38] and HSPGs (heparan sulfate proteoglycans). When CSP binds to low-sulfate HSPGs on dermal fibroblasts or endothelial cells, sporozoites transmigrate the host cells without parasitophorous vacuole formation. If CSP interacts with high-sulfate HSPGs on hepatocytes, it will be cleaved and supposed to expose the TSR (thrombospondin repeat) domain, and the binding of TSR domain to HSPGs leads to sporozoite invasion of hepatocytes [39, 40]. Once inside the final hepatocyte, a sporozoite is enclosed in a parasitophorous vacuole [3], which is separated from the lysosome to avoid degradation by the endocytic/lysosome system.

To survive and develop in the parasitophorous vacuole, the parasite has developed several strategies to suppress hepatocyte function while preventing cell death. For instance, cleaved CSP escapes from the parasitophorous vacuole into hepatocyte cytoplasm using its PEXEL domain [30]. Cleaved CSP that is translocated into the cytoplasm inhibits host cell protein synthesis by binding ribosomes, which might be beneficial for the development of the sporozoite [31]. CSP released into the cytoplasm possibly promotes parasite development through the suppression of NF-*κ*B [30]. Furthermore,* P. berghei* sporozoites infection inhibited hepatocyte apoptosis [41], and external HGF/cMET signaling is also involved in this process through upregulation of MAPK and PI3-kinase/Akt [42]. Very recently, Kaushansky et al. found that the majority of hepatocytes infected with wildtype but not attenuated liver-stage parasites can resist Fas-mediated apoptosis via an antiapoptotic mitochondrial protein [43]. Using protein lysate microarrays, they also found that hepatocyte regulatory pathways involved in cell survival (Bcl-2), proliferation, and autophagy (mTOR) were significantly perturbed by the* P. yoelii* sporozoite infection. Notably, the prodeath protein p53 was substantially decreased in infected hepatocytes, which allowed parasite survival [44].

Autophagy is a bulk degradation system that delivers cytoplasmic constituents and organelles into lysosomes for hydrolysis. It is originally thought to be essential for cell survival, development, and homeostasis, but growing evidence supported that autophagy could also restrict viral infections and the replication of intracellular bacteria and parasites [45]. Although autophagy was found to be involved in the transformation of sporozoites into the liver stage [46], the role of hepatocyte autophagy on the development of the pre-erythrocytic stage has not been reported. It is, therefore, interesting to investigate whether the sporozoite infection could induce hepatocyte autophagy and its effect on pre-erythrocytic stage development.

**Figure 2:**
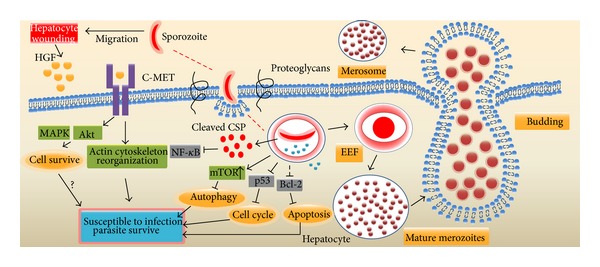
The manipulation of hepatocytes by sporozoites. Sporozoites transmigrate several hepatocytes prior to settling in a final cell. Transmigrated hepatocytes release HGF, which binds to the C-MET receptor, making the hepatocyte susceptible to infection and resistant to apoptosis by upregulation of MAPK, and Akt (left). The interaction of CSP with high levels of HSPGs triggers the cleavage of CSP and encapsulation of sporozoites in parasitophorous vacuoles. Cleaved CSP escapes from the parasitophorous vacuole into the cytoplasm, where it inhibits the NF-*κ*B activation and host protein synthesis. Sporozoite invasion upregulates mTOR and downregulates p53, and Bcl-2 which block autophagy, cell cycle progression and Apoptosis, respectively (middle). To avoid destruction by KCs and DCs during release from hepatocytes, merozoites bud from the hepatocytes in merosomes, which are covered with host cell-derived membranes, and PS exposure on the outer leaflet of the dying hepatocytes is blocked (right).

In addition to the period when sporozoites develop into merozoites in hepatocytes, merozoites also evade host defenses when they exit hepatocytes. To access the bloodstream, liver-stage merozoites must leave hepatocytes and cross both the Disse and sinusoid spaces, where they are vulnerable to be attacked by phagocytes including KCs and DCs. To avoid host cell defense mechanisms, merozoites bud from detached hepatocytes in merosomes [4, 47], which are covered with host cell-derived membranes [48]. During this process, the infected hepatocyte dies, but merozoites uptake Ca^2+^ and maintain low Ca^2+^ levels in the host cell to block the exposure of PS (phosphatidylserine) on the outer leaflet of the dying cells [4, 47]. Thus, dying hepatocytes avoid recognition by phagocytes, and merosomes are safely shielded from the hepatocytes. Merosomes eventually disintegrate inside pulmonary capillaries, which liberate merozoites into the bloodstream and for erythrocyte invasion [49] (Figure 2).

## 5. Concluding Remarks

Sporozoite infection elicits robust innate immune responses to limit its development into the erythrocytic stage. However, this parasite has evolved several escape strategies at each step of the liver-stage infection. For example, sporozoites could suppress the immune functions in KCs to ensure their safe passage through the sinusoidal cell layer of the liver. Once inside the hepatocyte, sporozoites could also inhibit the apoptosis of the infected hepatocyte to foster their development into EEFs, but they also induce host cell death after their release from the liver in merosomes. However, sporozoite challenge upregulates HO-1 (heme oxygenase-1), which promotes the development of the liver stage by inducing anti-inflammatory cytokines [50]. Although great progress has been made in recent years, some questions still remain. For instance, do molecules other than CSP escape from the PV to the cytoplasm and suppress hepatocyte functions? Does sporozoite infection induce hepatocyte autophagy? What is the effect of autophagy on pre-erythrocytic stage development? Answering these questions will not only help us to further understand the immune evasion strategies of sporozoites but will also provide us with novel targets for preventing malaria. For example, our previous study showed that preactivation of innate immune cells, such as KC, by individual TLRs agonists could significantly prevent the development of the pre-erythrocytic stage [28, 51].
